# A Triboelectric Nanogenerator Based on Sodium Chloride Powder for Self-Powered Humidity Sensor

**DOI:** 10.3390/nano11102657

**Published:** 2021-10-09

**Authors:** Zhuyu Ding, Ming Zou, Peng Yao, Zhiyuan Zhu, Li Fan

**Affiliations:** 1 College of Engineering and Technology, Southwest University, Chongqing 400715, China; dingzy@swu.edu.cn; 2 School of Electronic and Information Engineering, Southwest University, Chongqing 400715, China; zouming6289@email.swu.edu.cn (M.Z.); qw578592005@email.swu.edu.cn (P.Y.); zyuanzhu@swu.edu.cn (Z.Z.); 3 Ocean College, Faculty of Engineering, Zhejiang University, Hangzhou 316021, China

**Keywords:** triboelectric nanogenerator (TENG), sodium chloride powder, self-powered sensor, low-cost

## Abstract

Recently, the research of distributed sensor networks based on triboelectric technology has attracted extensive attention. Here, we reported a new triboelectric nanogenerator based on sodium chloride powder (S-TENG) to obtain mechanical energy. The polytetrafluoroethylene (PTFE) film and sodium chloride powder layer serve as the triboelectric pair. After testing and calculation, the internal resistance of S-TENG is 30 MΩ, and the output power of S-TENG (size: 6 cm × 6 cm) can arrive at the maximum value (about 403.3 µW). Furthermore, the S-TENG can achieve the open circuit voltage (*V_oc_*) of 198 V and short-circuit current (*I_sc_*) of 6.66 µA, respectively. Moreover, owing to the moisture absorption of sodium chloride powder, the S-TENG device also has the function of the humidity sensor. This work proposed a functional TENG device, and it can promote the advancement of self-powered sensors based on the TENG devices.

## 1. Introduction

Recently, owing to the progress needs of the Internet of things (IoT), various sensor technologies show numerous application prospects widely in the domain of the (IoT) [1,2,3]. As a significant part of the IoT, distributed sensor network has attracted the attention of academia and industry [4,5]. Often, distributed sensor networks consist of many sensors, but this poses new challenges to energy supply [6]. It is noteworthy that renewable energy generation is widely concerned, such as solar energy, ocean wave, temperature difference energy, wind and other green energy [7]. Compared with traditional fossil energy (oil, coal and natural gas), renewable energy has the characteristics of rich reserves, and is inexhaustible, and can reduce environmental pollution [8,9]. Therefore, harvesting technologies based on green renewable energy, such as electromagnetic power generation technology, piezoelectric power generation technology, photoelectric power generation technology and thermoelectric power generation technology, have exploded over the past few years. However, there are still many challenges in energy harvesting efficiency and use environment. In addition, the high preparation cost is also an important reason to hinder its application in distributed sensor networks [10]. In recent years, with the development of energy storage technology, distributed sensor network nodes usually provide power by electronics. However, the limited service life of the battery has brought a lot of replacement and maintenance work. Furthermore, this has an impact on the development of the Internet of things [11,12]. In addition, there will be environmental pollution problems. Therefore, the development of new power generation technology is necessary and meaningful.

In 2012, Professor Wang and his research group reported the triboelectric nanogenerator (TENG). The TENG device can convert low frequency and low amplitude mechanical energy into electrical energy output [13,14,15,16,17,18,19,20,21]. Furthermore, TENG devices exhibit an extensive application prospect in the fields of self-powered sensors, ocean wave energy and high-voltage power sources [22,23,24,25]. In addition, it has a profound and significant influence on the sustainable development of energy and environmental protection. The triboelectrification phenomenon can occur between most materials, and friction movement is everywhere in life [26,27]. Thus, the TENG devices have a wide range of preparation materials, and this also promotes the rapid development of TENG devices based on different triboelectric material combinations [28,29,30]. Up to now, TENG can gain almost all mechanical energy and convert it into electrical energy, such as ocean wave, breeze energy, human motion and other mechanical vibration energy in the form of low frequency [31,32,33,34,35]. In addition, TENG devices can respond to changes in the environment through changes in electrical output signals. Therefore, it is meaningful to develop a TENG device with a sensing function.

Here, we propose a novel triboelectric nanogenerator based on sodium chloride powder (S-TENG) to obtain mechanical energy. Furthermore, the S-TENG serves as the self-powered humidity sensor. It is noteworthy that sodium chloride is a kind of food material, which is non-toxic, pollution-free and rich in reserves. In addition, sodium chloride is easily soluble in water, which also creates conditions for material recycling. The polytetrafluoroethylene (PTFE) film and sodium chloride powder layer form the triboelectric pair. The conductive aluminum tape serves as the conductive electrode, and the glue section is used to paste triboelectric materials. From the results, the output power of S-TENG (size: 6 cm × 6 cm) can arrive at the maximum value (about 403.3 µW), and the internal resistance of S-TENG is 30 MΩ. Furthermore, the S-TENG can achieve the *V_oc_* of 198 V and *I_sc_* of 6.66 µA, respectively. Moreover, the S-TENG device can monitor environmental humidity.

## 2. Materials and Methods

**Figure 1:**
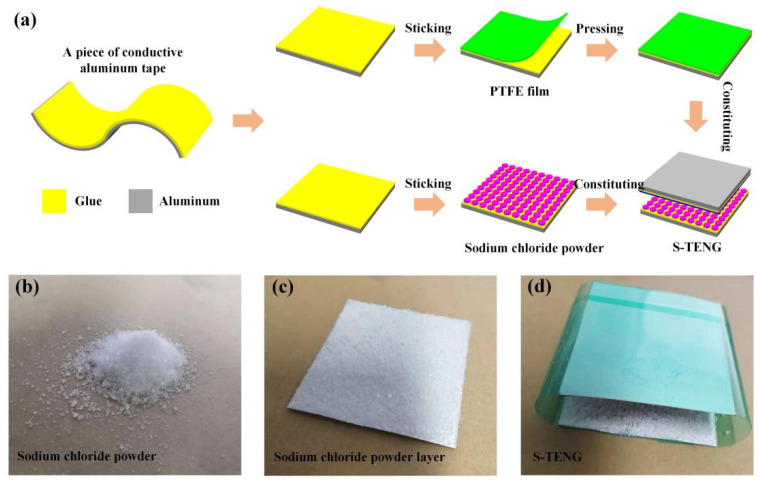
(**a**) The detailed preparation process of S-TENG using aluminum taper, PTFE film and sodium chloride powder. The photograph of (**b**) sodium chloride powder, (**c**) sodium chloride powder layer and (**d**) S-TENG.

In this design, the PTFE film (thickness: 120 µm) and sodium chloride powder layer form the triboelectric pair, and the aluminum foil serves as the conductive electrode. Figure 1a illustrates the detailed preparation process of S-TENG. Firstly, cut the aluminum tape into two pieces to fabricate the electrodes. It is worth noting that the conductive aluminum tape consists of aluminum and glue, and the glue can act as the adhesive to paste PTFE film and sodium chloride powder. Then, coat one piece of aluminum foil with PTFE film to form the PTFE/aluminum layer. As for another piece of aluminum tape, the sodium chloride powder is pasted on the glue surface to constitute the aluminum/sodium chloride powder layer. Finally, two triboelectric sections form the S-TENG device. In this work, we used the electrometer (Keithley 6517) to measure the electronic output, such as open circuit voltage (*V_oc_*), short-circuit current (*I_sc_*) and transfer charge. Additionally, we used mechanical vibration to provide the external force. Furthermore, the scanning electron microscope (SEM) images of PTFE film and sodium chloride powder layer were provided in Figures S1 and S2 of the Supporting Information.

## 3. Results and Discussion

**Figure 2:**
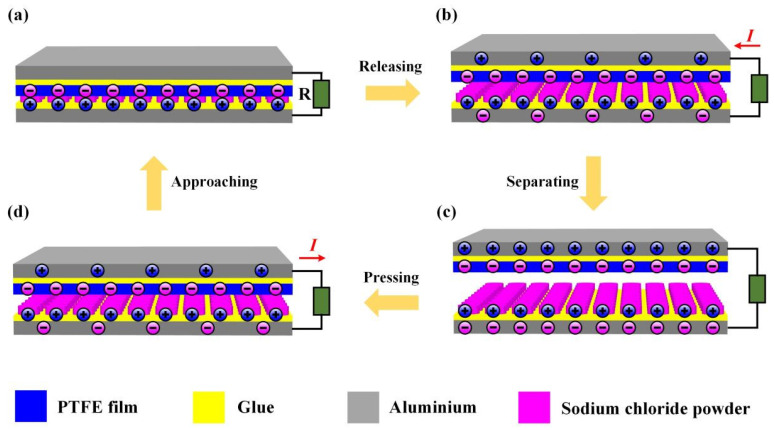
(**a**–**d**)The operating principle of S-TENG.

The S-TENG can work under vertical motion conditions, and the operating mechanism of S-TENG is shown in Figure 2. Generally, the PTFE film can obtain electrons from other triboelectric materials during the triboelectric process. Thus, when PTFE film contact with the sodium chloride powder layer, the PTFE film surface will obtain electrons, and the sodium chloride powder layer will lose the same amount of electrons due to the contact electrification mechanism, as shown in Figure 2a. Then, when the surfaces of the PTFE film and sodium chloride powder layer separate (Figure 2b), the top electrode of the S-TENG device will generate a positive charge, and the electrode at the bottom of the S-TENG will produce the same amount of negative charge. In addition, this can lead to the generation of pulse current in the external circuit. When the maximum separation distance reaches a certain value, the charge transfer between the two electrodes reaches the saturation state. Furthermore, the circuit will not produce pulse current, as shown in Figure 2c. when the PTFE film surface is close to the sodium chloride powder layer surface, the negative charge at the top electrode will be transferred to the bottom electrode, and a reverse pulse current will be formed, as shown in Figure 2d.

**Figure 3:**
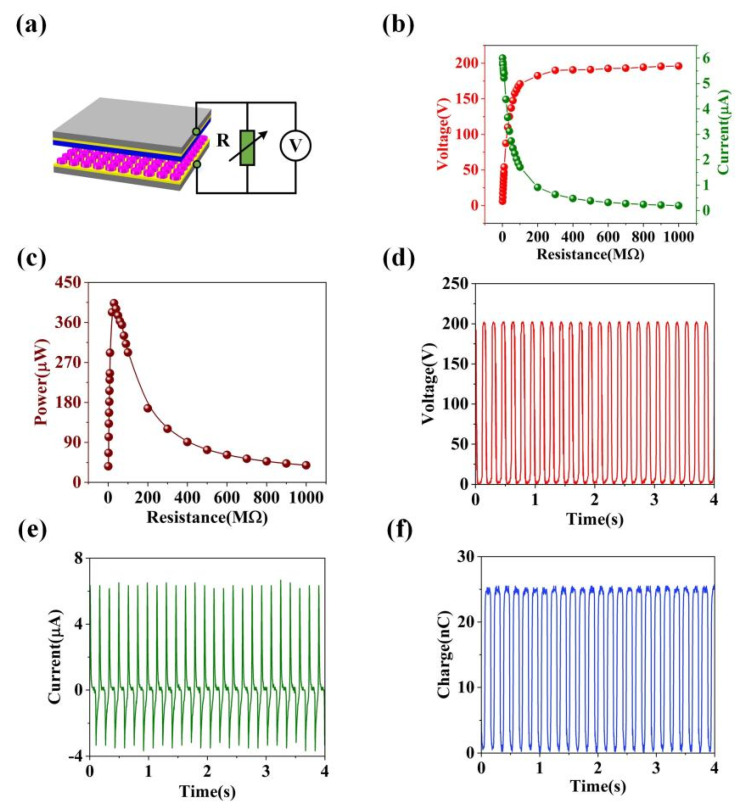
(**a**) The schematic diagram of electrical performance testing system about the S-TENG. (**b**,**c**) The relation between S-TENG output and resistance of loads. (**d**) *I_sc_*, (**e**) *V_oc_* and (**f**) charge transfer in the external circuit of S-TENG.

Moreover, we connect loads with different resistance values to S-TENG and measure the output performance (output voltage and current) of S-TENG, as shown in Figure 3a. The mechanical vibrator can provide an external force to drive the S-TENG. In addition, the motion parameters (such as vibration frequency and maximum separation distance) are set as 6 Hz and 5 mm, respectively. The size of the S-TENG device is about 6 cm × 6 cm. As is shown in Figure 3b, when the resistance of the load grows from 1 MΩ to 1 GM, the *V_oc_* of S-TENG will rise whereas the *I_sc_* of S-TENG will drop, which also indicates that TENG devices usually have high *V_oc_* and low *I_sc_*. Furthermore, we calculated the output power (*P*) of S-TENG through the relationship *P* = *UI*. In addition, Figure 3c describes the calculation results and relations. From the results, the S-TENG device can realize the maximum output power of 403.3 µW. Meanwhile, the internal resistance of S-TENG is 30 MΩ. Furthermore, the S-TENG can achieve the *V_oc_* of 198 V and *I_sc_* of 6.66 µA, respectively, as shown in Figure 3d,e. Figure 3f illustrates that the charge transfer in the external circuit can reach 25.5 nC.

**Figure 4:**
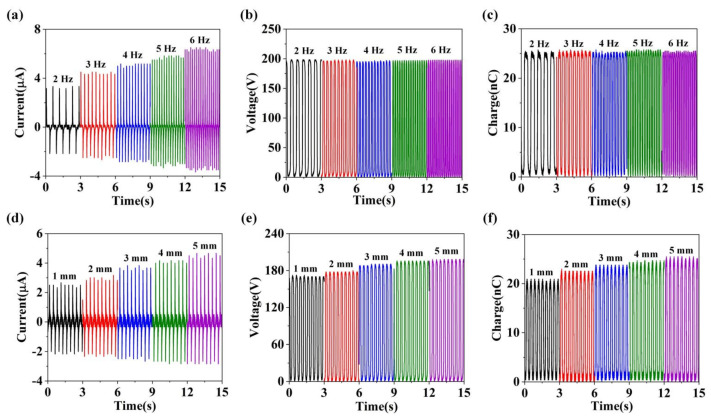
The (**a**) *I_sc_*, (**b**) *V_oc_* and (**c**) transfer charge of S-TENG under different operating frequencies. The (**d**) *I_sc_*, (**e**) *V_oc_* and (**f**) transfer charge of S-TENG under different maximum separation distance.

It is worthy to point out that the parameters of external excitation are the factors influencing the output characteristics of S-TENG. Therefore, we explored the influence of motion frequency and maximum separation distance on the electrical output of S-TENG. As shown in Figure 4a, when the working frequency rises from 2 Hz to 6 Hz, the *I_sc_* of S-TENG will grow from 3.33 µA to 6.5 µA. The reason for the increase of S-TENG is that the higher motion frequency is conducive to the rapid transfer of charges. As illustrated in Figure 4b,c, when the working frequency rises from 2 Hz to 6 Hz, the *V_oc_* of S-TENG will remain constant at about 198 V, and the transferred charge of S-TENG will also be unchanged at about 25.5 nC, which also indicates that the superiority of TENG devices in low-frequency motion energy harvesting. Moreover, the maximum separation distance between the PTFE film surface and sodium chloride powder layer surface can also influence the S-TENG electrical output. With the increase of the maximum separation distance (from 1 mm to 5 mm) shown in Figure 4c–e, the electrical output of S-TENG, such as *I_sc_*, *V_oc_* and transfer charge, will increase.

**Figure 5:**
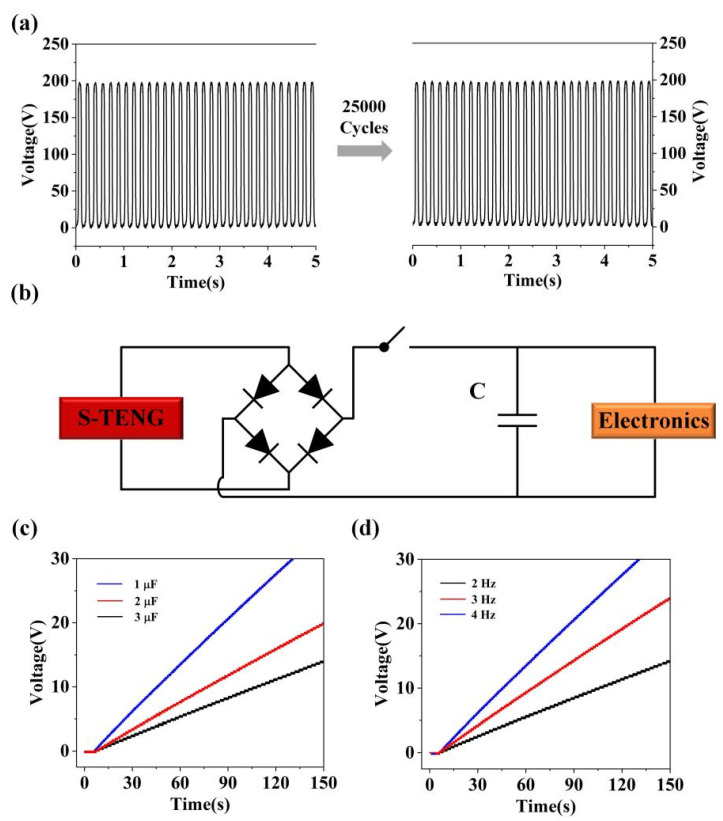
(**a**) The reliability test of S-TENG. (**b**) The schematic diagram of power management circuit based on the S-TENG. (**c**) The charging curve of S-TENG for different capacitors (from 1 µF to 3 µF) under the working frequency of 4 Hz. (**d**) The charging curve of S-TENG for 1 µF capacitor under different working frequencies (from 2 Hz to 4 Hz).

Moreover, considering the continuous work of S-TENG, we explored the electrical output of S-TENG under continuous operating conditions. Based on the results in Figure 5a, the S-TENG has good stability. Furthermore, we examine the charging effect of S-TENG with a power management circuit, as shown in Figure 5b. Here, we developed the relationship of S-TENG charging capacitors under different frequencies. Obviously, the higher the externally provided vibration frequency, the faster the rate of storing electric energy, as shown in Figure 5c. In addition, we also researched the influence of S-TENG charging different capacitors, as illustrated in Figure 5d. According to the experimental results, the larger the capacitor, the faster the charging speed. 

**Figure 6:**
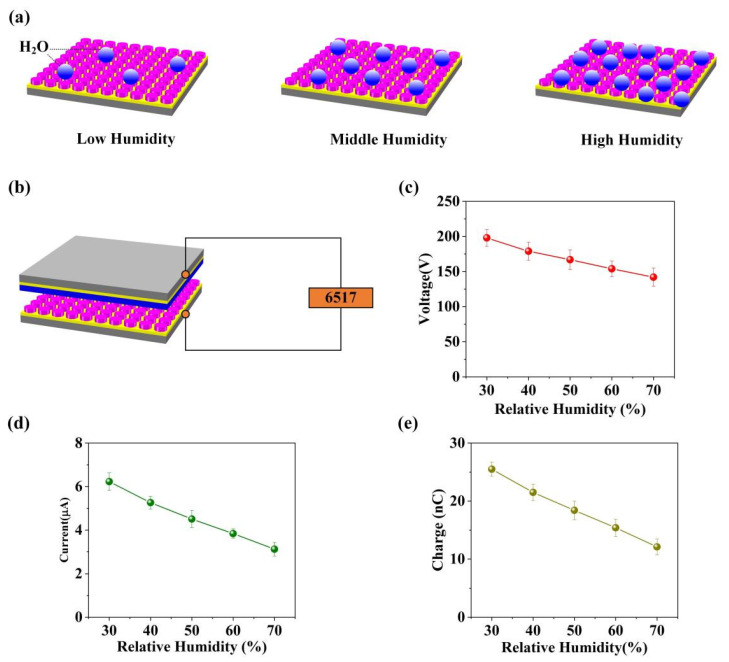
(**a**) The schematic illustration of the hygroscopicity for sodium chloride powder. (**b**) The relative humidity test system based on the S-TENG. (**c**–**e**) The electrical output of the S-TENG in different relative humidity conditions.

Often, TENG devices can convert moving mechanical energy into electrical energy during the contact and separation movement of triboelectric materials. In addition, the generated electrical signal is closely related to the influence of the working environment. Environmental factors will affect the electrical output signal produced by the TENG device, for example, relative humidity. It is noteworthy that sodium chloride powder has strong moisture absorption. Furthermore, this characteristic can make the S-TENG the self-powered humidity sensor by the electrical output signal change of the S-TENG device, as shown in Figure 6a,b. Specifically, the relative humidity will have a significant influence on the charge transfer of the TENG device. In this design, the sodium chloride powder plays the role of triboelectric material, and meanwhile, it is sensitive to relative humidity. Specifically, we measured the *V_oc_*, *I_sc_* and transferred charge of S-TENG under different relative humidity, as present in Figure 6c–e. According to the results, when the relative humidity rises, the electrical output (*V_oc_*, *I_sc_* and transferred charge) of S-TENG can grow, which indicates the S-TENG can monitor humidity changes.

## 4. Conclusions

In conclusion, we propose a novel triboelectric nanogenerator based on sodium chloride powder (S-TENG) to obtain mechanical energy. In addition, the S-TENG serves as the self-powered humidity sensor. It is noteworthy that sodium chloride is a kind of food material, which is non-toxic, pollution-free and rich in reserves. The PTFE film and sodium chloride powder layer form the triboelectric pair. The conductive aluminum tape is used as the conductive electrode, and the glue section is used to paste triboelectric materials. From the results, the output power of S-TENG (size: 6 cm × 6 cm) can arrive at the maximum value (about 403.3 µW). Furthermore, the S-TENG can achieve the *V_oc_* of 198 V and *I_sc_* of 6.66 µA, respectively. Moreover, the S-TENG device can monitor environmental humidity.

## Data Availability

Some or all data, models, or code generated or used during the study are proprietary or confidential in nature and may only be provided with restrictions.

## Supplementary Materials

The following are available online at https://www.mdpi.com/article/10.3390/nano11102657/s1, Figure S1: The SEM images of PTFE before and after 24 k impact cycles. Figure S2: The SEM image of the NaCl powder layer surface.
