# Supplementary material for: A Triboelectric Nanogenerator Based on Sodium Chloride Powder for Self-Powered Humidity Sensor
Source: Nanomaterials (Basel). 2021 Oct 9;11(10):2657. doi: 10.3390/nano11102657 (PMC8538726; doi:10.3390/nano11102657)
Supplement: Supplementary file 1 [file nanomaterials-11-02657-s001.zip › nanomaterials-1404285-supplementary.pdf]

nanomaterials

Supplementary Materials

A Triboelectric Nanogenerator Based on Sodium Chloride Powder for Self-powered Humidity Sensor

Zhuyu Ding [1], Ming Zou [2], Peng Yao [2], Zhiyuan Zhu [2,3] and Li Fan [2,*]

[1] College of Engineering and Technology, Southwest University, Chongqing 400715, China; dingzy@swu.edu.cn
[2] School of Electronic and Information Engineering, Southwest University, Chongqing 400715, China; zouming6289@email.swu.edu.cn (M.Z.); qw578592005@email.swu.edu.cn (P.Y.); zyuanzhu@swu.edu.cn (Z.Z.)
[3] Ocean college, Faculty of Engineering, Zhejiang University, Zhejiang 316021, China
* Correspondence: fanli123@swu.edu.cn

In this work, we provide the surface morphology of the pristine and the PTFE films after the 24k impact cycles, as shown in Fig. S1(a, b). Furthermore, we also provided the SEM image of NaCl powder layer surface sample, as shown in Fig. S2.

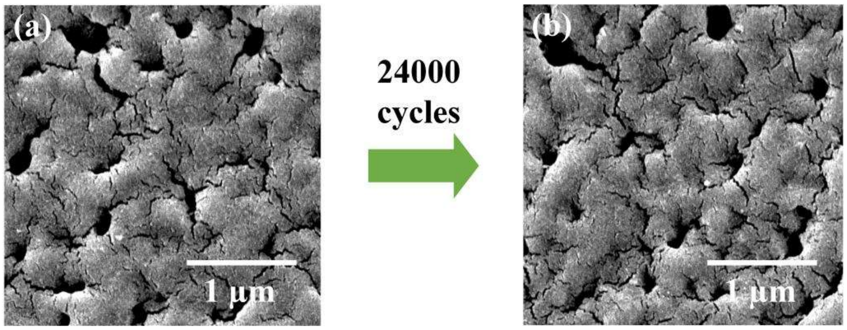

Figure S1. (**a**,**b**). The surface morphology of the pristine and the PTFE films after the 24k impact cycles.

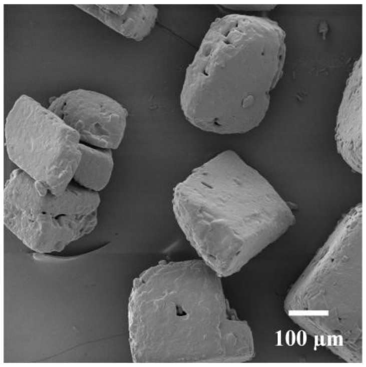

Figure S2. The SEM image of NaCl powder layer surface.
